# Outcomes of Watch-and-Wait Versus Abdominoperineal Resection in Lower Rectal Adenocarcinoma Post Neoadjuvant Therapy: An Iraqi Cohort Study

**DOI:** 10.7759/cureus.67955

**Published:** 2024-08-27

**Authors:** Aqeel S Mahmood, Osama Jalal Fakhir, Haider A Ahmed, Manwar Abdulelah Alnaqqash, Tahseen Alrubaei, Wieeam Abdulfattah Saleh, Ahmed A Alkadir, Ahmed Zuhair Alsammarraie, Forat Yahya Mohsin, Ahmed A Shakir, Yesor Jamal Albadri, Mustafa Ismail

**Affiliations:** 1 Department of Surgery, University of Baghdad, Baghdad, IRQ; 2 Department of Surgical Oncology, Oncology Teaching Hospital, Baghdad, IRQ; 3 Department of Surgery, Iraqi Board for Medical Specializations, Baghdad, IRQ; 4 Department of Surgery, Medical City, Baghdad Teaching Hospital, Baghdad, IRQ; 5 Department of Surgery, Baghdad Teaching Hospital, Baghdad, IRQ; 6 Department of Surgery, College of Medicine, University of Baghdad, Baghdad, IRQ

**Keywords:** neoadjuvant chemoradiotherapy, watch-and-wait, complete clinical response, colorectal adenocarcinoma, abdominoperineal resection

## Abstract

Background: Rectal malignancy ranks among the most prevalent malignancies in humans. Neoadjuvant chemoradiotherapy (nCRT) is advocated as the standard treatment for locally advanced rectal cancer. In patients who achieve complete clinical response (cCR), successive surgical intervention may result in favorable immediate and long-lasting results; however, it may be associated with decreased quality of life. This study aims to evaluate the incidence of local recurrence in rectal adenocarcinoma between patients who underwent a watch-and-wait approach and those who underwent abdominoperineal resection following the achievement of a cCR after nCRT.

Methods: This is an analytic cohort study that included 68 patients and was conducted in Baghdad Teaching Hospital/Medical City, Baghdad. The data were collected from the 1st of April 2021 to the 1st of October 2023. All patients with stage II and III rectal adenocarcinoma who achieved cCR after receiving nCRT were included in the study.

Results: There was no statistically significant difference between the two study groups regarding non-regrowth disease-free survival (p-value = 0.708). Cox-regression multivariate analysis revealed that baseline T stage and serum carcinoembryonic antigen (CEA) were significantly associated with locoregional failure.

Conclusion: The present study reveals that implementing the watch-and-wait strategy had the benefit of avoiding major surgery, stoma, and their complications without coming at the cost of reduced locoregional recurrence.

## Introduction

In Iraq, there has been a notable rise in the proportion of colorectal cancer cases in relation to the total number of cancer cases. Specifically, this ratio has escalated from 3.69% in the year 2000 to 6.5% by the year 2019. Additionally, the mortality rate attributed to colorectal cancer has also seen an increase, moving from 1.25 per 100,000 individuals in the year 2010 to 1.77 per 100,000 individuals by the year 2019 [1-3]. This upward trend in both the incidence and mortality rates of colorectal cancer highlights the growing significance of this disease within the Iraqi population and underscores the need for enhanced screening, prevention, and treatment strategies to address this public health concern.

Rectal cancer arises from genetic and epigenetic changes in the colonic and rectal epithelium, leading to the transformation of colorectal adenomas into invasive carcinomas. These mutations are categorized as sporadic, inherited, or familial [4]. Risk factors for rectal cancer include advancing age, hereditary conditions such as Lynch syndrome and familial adenomatous polyposis, and lifestyle factors like tobacco smoking and alcohol consumption [5]. Additionally, dietary habits, particularly high consumption of red and processed meats, low intake of fiber, fruits, and vegetables, and insufficient vitamin D levels, are associated with elevated risks. Other factors such as cholecystectomy and chronic inflammation due to inflammatory bowel disease also play a role in increasing the risk of colorectal cancer [6]. A multidisciplinary approach that includes surgeons, gastroenterologists, medical oncologists, radiologists, pathologists, nursing staff, physiotherapists, psychologists, and radiation oncologists is required for optimal treatment of patients with rectal cancer [7].

Although surgical management of rectal cancer emphasizes complete tumor excision, total mesorectal excision (TME) and similar techniques have reduced the rate of local recurrence and improved survival. However, there is considerable morbidity associated with radical surgery, including impaired continence. Organ-preserving strategies are also gaining increased attention for these reasons. One such strategy is the watch-and-wait approach, which has emerged to be a viable option for patients who achieve a complete clinical response (cCR) after neoadjuvant chemoradiotherapy (nCRT). This spares the patient from possible surgical complications without affecting oncological results and hence becomes a critical consideration in the multidisciplinary management of rectal cancer [7].

This study aims to compare the local re-growth of rectal adenocarcinoma between the watch-and-wait strategy and abdominoperineal resection for patients who achieve cCR response after nCRT.

## Materials and methods

An analytic cohort design has been chosen for this study that has been conducted in Baghdad Teaching Hospital/Medical City, Baghdad. The data were collected from the 1st of April 2021 to the 1st of October 2023. All patients with stage II and III rectal adenocarcinoma who achieved cCR after receiving nCRT were included in the study. Exclusion criteria included prior transanal excision (stage I colorectal carcinoma), the coexistence of a second primary malignant disease within a five-year period, and patients not achieving cCR to nCRT (partial or no response) to maintain a homogeneous study population. The provision of patients with partial or nonappearance of response would have led to the inclusion of variability in treatment results, which could have developed confounding existence for the results so that it becomes harder to elucidate a distinct comparison between the two management strategies; thus, for providing valid and reliable findings of the study, we have included only those who obtained cCR. Among 84 cases, 68 patients were selected, while 16 patients were excluded due to reasons mentioned in the exclusion criteria (Tables 1, 2).

**Table 1:** Basic characteristics of the studied sample.

Variable	Approach		P-value
	Surgery	Watchful waiting	
Age			
<40 years	13	5	0.477
	26.0%	27.8%	
40-60 years	29	7	
	58.0%	38.9%	
>60 years	8	6	
	16.0%	33.3%	
Mean ± SD	48.6 ± 13.0	51.2 ± 13.6	
BMI			
Normal weight	24	5	0.531
	48.0%	27.8%	
Overweight	5	6	
	10.0%	33.3%	
Obese	21	7	
	42.0%	38.9%	
Mean ± SD	28.1 ± 6.3	29.1 ± 5.1	
Gender			
Male	24	8	1.000
	48.0%	44.4%	
Female	26	10	
	52.0%	55.6%	
Smoking history			
Yes	24	8	1.000
	48.0%	44.4%	
No	26	10	
	52.0%	55.6%	
Family history			
Positive	33	7	0.055
	66.0%	38.9%	
Negative	17	11	
	34.0%	61.1%	

**Table 2:** Tumor characteristics of the studied sample.

Variable	Approach		P-value
	Surgery	Watchful waiting	
Histological grade			
Well-differentiated	0	4	0.001
	0.0%	22.2%	
Moderately differentiated	25	11	
	50.0%	61.1%	
Poorly differentiated	25	3	
	50.0%	16.7%	
Pretreatment T stage			
T2	20	8	0.785
	40.0%	44.4%	
T3	30	10	
	60.0%	55.6%	
Pretreatment N stage			
N0	1	7	<0.001
	2.0%	38.9%	
N1	20	8	
	40.0%	44.4%	
N2	29	3	
	58.0%	16.7%	
Serum carcinoembryonic antigen (CEA)			
Mean ± SD	9.7 ± 3.7	7.2 ± 3.4	0.017
Height from the anal verge			
Mean ± SD	3.9 ± 1.2	5.1 ± 1.5	0.001

cCR in rectal cancer was defined as the achievement using a judicious set of criteria from multiple reassessment methods. A digital rectal examination confirmed that there is no longer a palpable tumor or irregularity at the site of the primary tumor. Pelvic MRI findings were also very important, in which diffusion-weighted imaging did not show a hyperintense signal at the site of the tumor, while T2-weighted imaging represented a hypointense signal suggesting significant fibrosis without residual tumor. In support of this diagnosis, an endoscopic examination identified a flat scar or whitening at the site of tumors, and multi-point biopsy pathology confirmed the complete absence of viable tumor cells. Serum carcinoembryonic antigen levels were also measured, which were within normal limits and without any significant rise post-nCRT; it ruled out active disease. Lastly, comprehensive imaging by CT or MRI excluded distant metastasis and hence established that the cancer had not spread beyond the local site.

After the achievement of cCR, patients were divided into two groups. The first group included 50 patients who underwent surgery, while the second group included 18 patients who underwent the watchful waiting approach. The chosen approach was determined by the consultant oncological surgeon according to the patient’s criteria (patient’s will, socioeconomic status, compliance to treatment, tumor grade, stage, family history, and anesthetic fitness). For instance, patients who were determined to be unfit for surgical intervention or those who refused surgery were assigned to undergo watchful waiting. All patients underwent follow-up every three months in the first two years and every six months in the third year.

Local regrowth is defined as recurrence at the primary site in the rectum following a cCR to neoadjuvant treatment. This differs from the entity of local recurrence, which can arise in surrounding tissues or lymph nodes.

Data entry was done using Microsoft Excel 2019 (Microsoft Corporation, Redmond, WA). Data were recorded into different quantitative and qualitative variables for the purpose of analysis. Analysis was done using SPSS version 26 (IBM Corp., Armonk, NY). Data were summarized using measures of frequency (mean), dispersion (standard deviation), tables, and graphs. A two-tailed p-value of less than or equal to 0.05 was assigned as a criterion for declaring statistical significance.

Verbal and written consent has been obtained from all participants before data collection. An official letter of approval has been obtained from the Scientific Committee of the Scientific Council of General Surgery - Iraqi Board for Medical Specializations (Approval No.: 1053).

## Results

**Figure 1:**
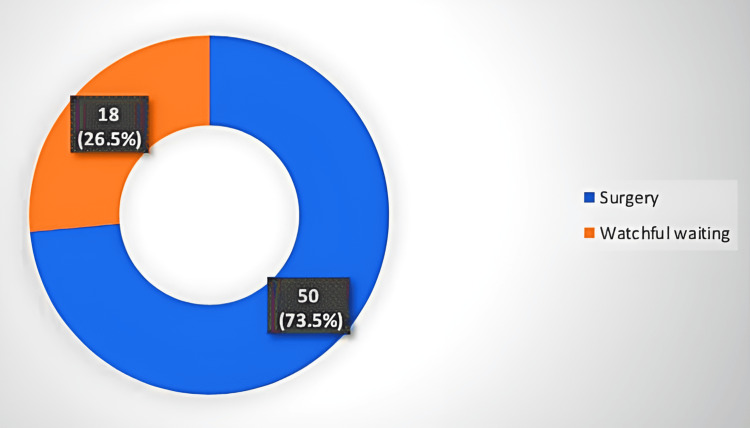
Distribution of patients according to the treatment approach.

Among 68 patients, 50 (73.5%) patients underwent surgery, while 18 (26.5%) underwent watchful waiting, as shown in Figure 1. No significant difference between the two study groups was detected regarding age, BMI, gender, smoking history, and family history. A statistically significant difference was detected among the two study groups regarding histological grade, baseline N stage, serum carcinoembryonic antigen (CEA), and height from the anal verge.

**Figure 2:**
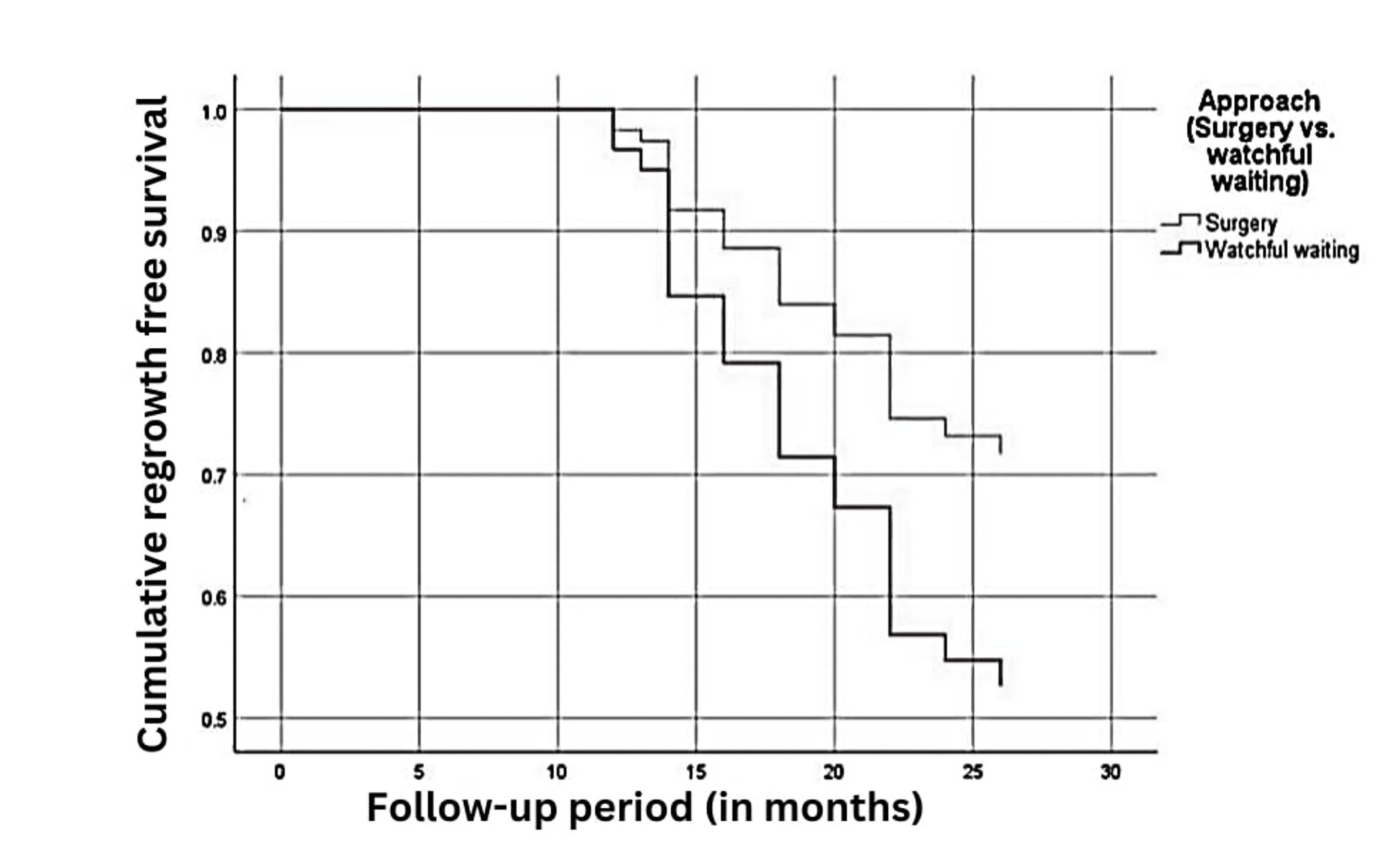
Non-regrowth disease-free survival of both study groups.

Local regrowth was detected in 17/50 (34%) cases of the surgery group and 8/18 (44.4%) cases of the watch-and-wait group, as shown in Table 3. Survival analysis revealed no statistically significant difference between the two study groups regarding non-regrowth disease-free survival (p-value = 0.708 by log-rank test), as shown in Figure 2. Cox-regression multivariate analysis revealed that baseline T stage and serum CEA were significantly associated with locoregional failure, as shown in Table 4.

**Table 3:** Non-regrowth disease-free survival in both study groups.

Non-regrowth disease-free survival	Approach	
	Watch-and-wait (N = 18)	Surgery (N = 50)
Locoregional regrowth	8	17
	44.4%	34.0%
No locoregional recurrence	10	33
	55.6%	66.0%

**Table 4:** Multivariate analysis of factors associated with locoregional failure.

Variable	Hazard ratio	95% CI		P-value
		Lower	Upper	
Approach				
Surgery	Ref.	Ref.	Ref.	Ref.
Watchful waiting	1.928	0.608	3.111	0.265
Age				
	1.009	0.977	1.042	0.580
Pretreatment T stage				
T2	Ref.	Ref.	Ref.	Ref.
T3	2.659	1.053	6.716	0.039
Pretreatment N stage				
Stage N0	Ref.	Ref.	Ref.	Ref.
Stage N1	0.963	0.259	3.581	0.955
Stage N2	0.494	0.103	2.363	0.377
Histological grade				
Well-differentiated	Ref.	Ref.	Ref.	Ref.
Moderately differentiated	0.993	0.179	5.513	0.994
Poorly differentiated	1.700	0.251	11.528	0.587
Baseline serum carcinoembryonic antigen (CEA)				
	1.172	1.028	1.336	0.018

## Discussion

The United States National Comprehensive Cancer Network endorses total neoadjuvant therapy as the preferred approach for the treatment of locally advanced rectal cancer, reflecting the latest standard of care. It incorporates both chemotherapy and radiation before surgery in such a sequence that tumor response may be achieved in the most optimal way, while oncological outcomes in patients may be maximized [8]. This strategy provides multiple advantages, such as reducing tumor volume, lowering the pathological stage, enhancing the likelihood of successful surgical resection or sphincter preservation, and significantly decreasing the rates of local recurrence and metastasis.

In patients who achieve cCR, subsequent surgical intervention can lead to positive outcomes, both immediate and long-term results for those diagnosed with rectal cancer. However, this approach might not always be the most beneficial, particularly for older individuals with multiple coexisting medical conditions. Additionally, complications arising from extensive surgical procedures, such as low anterior resection syndrome and a high rate of stoma creation, can significantly reduce the quality of life for patients [9].

Research indicates that patients who obtained complete remission following radical surgery can experience postoperative complications, including anastomotic leakage, where the surgical join leaks, with incidence rates between 1.8% and 20% [10]. Such complications can significantly impact the patient's quality of life and may result in stoma stricture, cancer recurrence, and severe functional limitations. Consequently, both surgeons and patients often prefer to avoid the creation of a stoma [11]. Due to these concerns, Habr-Gama et al. [12] introduced the "watch and wait" approach in 2004 as an alternative to surgical intervention for patients who achieve clinical complete remission after nCRT.

This is the first study in Iraq aiming to compare the local regrowth of the watch-and-wait approach with that of surgery for patients who achieve cCR after nCRT. In the present study, local regrowth was detected in 34% of cases of the surgery group and 44.4% of cases of the watch-and-wait group. Survival analysis revealed no significant difference among the two groups. This indicates that 55.6% of patients in the watch-and-wait group avoided major surgery.

Renehan et al. [13], Wang et al. [14], and Al-Najami et al. [15] reported that the watch-and-wait was non-inferior to the surgery group and had local regrowth rates of 17%, 14.9%, and 26%, respectively. This variation might be a result of different follow-up durations, surveillance intensity, and sample size. In contrast, Yu et al.’s meta-analysis [16] revealed that the watch-and-wait group had a greater likelihood of local recurrence compared to the total mesorectal excision group. However, both groups had equal overall survival rates. Moreover, the research revealed that by using physical assessment and salvage therapy, the watch-and-wait technique not only reduces surgical trauma and ensures quality of life but also attains favorable clinical outcomes. Therefore, the watch-and-wait approach was suggested as a potentially advantageous approach for rectal cancer with cCR status. It is noteworthy to mention that the median follow-up period in the current study was 36 months (three years). A systematic review conducted by Dattani et al. [17] found that the majority of locoregional failures took place during the first three years of monitoring, with two-thirds happening within the first year. These worrisome statistics emphasize the urgent need for strict early monitoring systems to prevent the recurrence of tumors. It is recommended by best practice guidelines that patients have regular examinations including digital rectal examination, endoscopy, CEA level testing, and MRI evaluation every eight to 12 weeks in the first three years.

Our study detected the majority of locoregional recurrences in this important period, and the case therefore reinforces the need for serial review, especially during the early post-treatment years. Although Dattani et al. emphasized the overall time point at which the majority of locoregional regrowth occurred, our work provides actual numbers for the patient group in Iraq and considerably adds regional information to the global snapshot from rectal cancer treatment studies. Furthermore, our results emphasize the possibility of non-surgical management measures in the avoidance of radical surgery without impairing locoregional control and therefore add depth to the findings of Dattani et al. [17]. Our study not only complements the results found in other sources but also adds a unique perspective on the effectiveness of non-surgical management strategies in a specific population.

After the three-year period, the follow-up can be rendered more lenient by switching to biannual examinations [18]. In patients with local regrowth, the success rate of salvage surgical interventions is as high as 95.4% [19]. The research conducted by Smith et al. [20] revealed that 20% of the 113 patients in the watch-and-wait group had local recurrence. However, successive salvage procedures were effectively performed, highlighting the strong practicality of the watch-and-wait technique for patients with local recurrence.

Regarding other factors associated with local recurrence, the present study found that a higher T stage was significantly associated with local recurrence. This is not surprising given that studies found that baseline T stage was the most important predictor for local recurrence. The meta-analysis by Chadi et al. [21] revealed that in patients who undergo watch and wait, the cumulative incidence of local regrowth was 19% for stage cT1 and cT2 tumors, 31% for cT3, and 37% for cT4. Concerning age, the current study has found no association of age with locoregional recurrence. This is in discordance with Steele et al. [22], who reported that an age <40 years was significantly associated with locoregional recurrence, whereas the study by Mima et al. [23] reported that age >70 years was an independent risk factor for local recurrence of stage II colorectal carcinoma.

The present study has also revealed that nodal staging was not associated with recurrence. This is in concordance with Hayes et al. [24], who found no association between increasing nodal count and recurrence-free survival, as the nodal stage was only linked with decreasing overall survival.

Clinically, the fact that nodal staging was unlinked to recurrence suggests that nodal status is perhaps not such a critical factor to be predicted in locoregional recurrence, as thought before. The number of nodes removed was shown not to be significantly associated with recurrence-free survival by Hayes et al., and this result is compatible with such findings. This may have a bearing on follow-up strategies, where greater emphasis could be placed on other factors such as tumor characteristics and molecular markers rather than nodal staging alone in considering the risk of recurrence. However, our study was limited by a small sample size; therefore, studies in larger cohorts are required to confirm these findings and enable possible revisions of the guidelines regarding post-treatment surveillance.

Moreover, histological grade was also not associated with locoregional recurrence. This is in discordance with Cho et al. [25], who found that the histological grade was the only influential factor in determining the survival time after colorectal cancer resection, as patients diagnosed with poorly differentiated carcinoma often had a recurrence and succumbed to the disease within three years after colon resection. The lack of significant association between locoregional recurrence and age, nodal stage, and histopathological grade can be attributed to the limited sample size, as this study was designed to examine the difference in recurrence-free survival among patients who underwent surgery and those with a watch-and-wait approach.

Serum CEA was also found to be a predictor of local regrowth. This is in concordance with Lin et al. [26], who concluded that serum CEA was an independent predictor for locoregional recurrence-free survival. However, Ramphal et al. [27] reported that no significant association was found between an elevated preoperative serum CEA and locoregional recurrence, but it was significantly associated with systemic recurrence. Nodal status was not found to be associated with locoregional recurrence. However, this finding may be due to the small sample size of the present study and a larger sample would probably find a positive association.

Clinically, it may have very significant implications for local regrowth, given the findings that serum CEA can predict it in the post-treatment management of patients diagnosed with rectal carcinoma. This would mean earlier detection of regrowth, allowing timely intervention that might improve patient outcomes. This contrasts with the report by Ramphal et al., wherein serum CEA was associated with systemic rather than locoregional recurrence, signifying that the follow-up policy is personalized with an emphasis on locoregional as well as systemic surveillance for patients with high serum CEA. This further justifies the incorporation of measurement of serum CEA in post-treatment monitoring protocols to properly stratify patients on the basis of their risk of recurrence.

In this respect, it proves that the watch-and-wait strategy is one of the valid non-surgical alternatives in patients with rectal adenocarcinoma who have undergone nCRT and achieved a cCR. We can conclude that this strategy avoids morbidity from major surgical interventions like abdominoperineal resection without an increased risk for locoregional recurrence. Although surgery remains integral to the management of rectal cancer, mostly in patients who have failed to achieve a cCR, the "watch-and-wait" strategy offers an important alternative that helps to preserve the quality of life for suitably selected patients. Results from this study underline the importance of individual treatment planning and rigorous follow-up protocols if optimum results are to be achieved in patients with locally advanced rectal cancer.

The study has several limitations that must be considered. First, the sample size is small, involving only 68 patients, thereby setting a potential limitation to the generalizability of such findings. Another limitation of this study is the follow-up duration; it is only three years, which, though an acceptable duration, requires a long-term follow-up for both treatment policies to understand a complete sense of their long-term outcomes and recurrence rates. Studies with larger multicentric cohorts and with longer follow-ups have to be done in the future to validate these results and provide more solid evidence to guide clinical practice in the management of rectal adenocarcinoma.

## Conclusions

The present study demonstrated that patients opting for the watch-and-wait strategy had the benefit of avoiding the morbidity of significant surgery with a stoma and its complications without any cost to reduce locoregional recurrence. Further multi-centric studies with larger sample sizes are recommended to validate our results. Based on our experience, we recommend the implementation of a scoring system for patients who make good candidates for the watch-and-wait strategy. The scoring system is recommended to include (age, sex, grade, stage, family history, and serum CEA). More studies need to shed light upon patients aged <60 years on whom surgical excision and stoma pose a more negative impact on the quality of life. Although the present study found that the watch-and-wait approach was non-inferior to surgical intervention in patients with colorectal cancer, this strategy is better implemented at tertiary centers where there is a strict follow-up of a multidisciplinary team of consultant surgeons, pathologists, radiologists, gastroenterologists, radiotherapists, and oncologists, especially in the first three years.

## References

[1] Alshewered AS, Al-Naqqash MA (2019). Rectal cancer and chemoradiation in Iraq: systematic review and meta-analysis. J Coloproctol.

[2] Dhahir NK, Noaman AA (2021). A comparative study of colorectal cancer based on patient’s age. J Fac Med Baghdad.

[3] Ibrahem S, Ahmed H, Zangana S (2022). Trends in colorectal cancer in Iraq over two decades: incidence, mortality, topography and morphology. Ann Saudi Med.

[4] Huang Z, Yang M (2022). Molecular network of colorectal cancer and current therapeutic options. Front Oncol.

[5] (2024). Centers for Disease Control and Prevention. Colorectal cancer risk factors. https://www.cdc.gov/colorectal-cancer/risk-factors/index.html.

[6] Beaugerie L, Carrat F, Nahon S (2018). High risk of anal and rectal cancer in patients with anal and/or perianal Crohn’s disease. Clin Gastroenterol Hepatol.

[7] Keller DS, Berho M, Perez RO, Wexner SD, Chand M (2020). The multidisciplinary management of rectal cancer. Nat Rev Gastroenterol Hepatol.

[8] Benson AB, Venook AP, Adam M (2024). NCCN Guidelines® Insights: rectal cancer, version 3.2024. J Natl Compr Canc Netw.

[9] Hughes DL, Cornish J, Morris C (2017). Functional outcome following rectal surgery—predisposing factors for low anterior resection syndrome. Int J Colorectal Dis.

[10] Paulinus JA (2021). A bioelectronic approach to post-surgical anastomotic leakage diagnosis. https://stax.strath.ac.uk/concern/theses/73666480p.

[11] Tan S, Gao Q, Cui Y, Ou Y, Huang S, Feng W (2023). Oncologic outcomes of watch-and-wait strategy or surgery for low to intermediate rectal cancer in clinical complete remission after adjuvant chemotherapy: a systematic review and meta-analysis. Int J Colorectal Dis.

[12] Habr-Gama A, São Julião GP, Vailati BB (2019). Organ preservation in cT2N0 rectal cancer after neoadjuvant chemoradiation therapy: the impact of radiation therapy dose-escalation and consolidation chemotherapy. Ann Surg.

[13] Renehan AG, Malcomson L, Emsley R (2016). Watch-and-wait approach versus surgical resection after chemoradiotherapy for patients with rectal cancer (the OnCoRe project): a propensity-score matched cohort analysis. Lancet Oncol.

[14] Wang QX, Zhang R, Xiao WW (2021). The watch-and-wait strategy versus surgical resection for rectal cancer patients with a clinical complete response after neoadjuvant chemoradiotherapy. Radiat Oncol.

[15] Al-Najami I, Jones HJ, Dickson EA, Muirhead R, Deding U, James DR, Cunningham C (2021). Rectal cancer: watch-and-wait and continuing the rectal-preserving strategy with local excision for incomplete response or limited regrowth. Surg Oncol.

[16] Yu G, Lu W, Jiao Z, Qiao J, Ma S, Liu X (2021). A meta-analysis of the watch-and-wait strategy versus total mesorectal excision for rectal cancer exhibiting complete clinical response after neoadjuvant chemoradiotherapy. World J Surg Oncol.

[17] Dattani M, Heald RJ, Goussous G (2018). Oncological and survival outcomes in watch and wait patients with a clinical complete response after neoadjuvant chemoradiotherapy for rectal cancer: a systematic review and pooled analysis. Ann Surg.

[18] Cerdán-Santacruz C, Vailati BB, São Julião GP, Habr-Gama A, Perez RO (2022). Watch and wait: why, to whom and how. Surg Oncol.

[19] Fernandez LM, São Julião GP, Figueiredo NL (2021). Conditional recurrence-free survival of clinical complete responders managed by watch and wait after neoadjuvant chemoradiotherapy for rectal cancer in the International Watch & Wait Database: a retrospective, international, multicentre registry study. Lancet Oncol.

[20] Smith JJ, Chow OS, Gollub MJ (2015). Organ preservation in rectal adenocarcinoma: a phase II randomized controlled trial evaluating 3-year disease-free survival in patients with locally advanced rectal cancer treated with chemoradiation plus induction or consolidation chemotherapy, and total mesorectal excision or nonoperative management. BMC Cancer.

[21] Chadi SA, Malcomson L, Ensor J (2018). Factors affecting local regrowth after watch and wait for patients with a clinical complete response following chemoradiotherapy in rectal cancer (InterCoRe consortium): an individual participant data meta-analysis. Lancet Gastroenterol Hepatol.

[22] Steele SR, Park GE, Johnson EK, Martin MJ, Stojadinovic A, Maykel JA, Causey MW (2014). The impact of age on colorectal cancer incidence, treatment, and outcomes in an equal-access health care system. Dis Colon Rectum.

[23] Mima K, Kurashige J, Miyanari N (2020). Advanced age is a risk factor for recurrence after resection in stage II colorectal cancer. In Vivo.

[24] Hayes IP, Milanzi E, Gibbs P, Faragher I, Reece JC (2022). Is increasing nodal count associated with improved recurrence-free and overall survival following standard right hemicolectomy for colon cancer?. J Surg Oncol.

[25] Cho YB, Chun HK, Yun HR, Kim HC, Yun SH, Lee WY (2009). Histological grade predicts survival time associated with recurrence after resection for colorectal cancer. Hepatogastroenterology.

[26] Lin Y, Liu S, Hong L, Shao L, Wu J (2022). Postoperative locoregional recurrence pattern and treatment management of stage pT4 sigmoid colon cancer: a retrospective cohort study. Radiat Oncol.

[27] Ramphal W, Boeding JR, van Iwaarden M, Schreinemakers JM, Rutten HJ, Crolla RM, Gobardhan PD (2019). Serum carcinoembryonic antigen to predict recurrence in the follow-up of patients with colorectal cancer. Int J Biol Markers.

